# Unmute the Organization Through Serious Play

**DOI:** 10.3389/fpsyg.2020.607919

**Published:** 2021-01-13

**Authors:** Frode Heldal, Erlend Dehlin, Torild Alise Oddane

**Affiliations:** Faculty of Economics, Trondheim Business School, Norwegian University of Science and Technology, Trondheim, Norway

**Keywords:** action research, serious play, replicability, unmuting, OU

## Abstract

In this article, we sketch up an action research process designed to give voice to those who traditionally have not had a voice in organizations. In particular, the research process was structured around “serious play” and designed as a talk show, where researchers played parts, including a talk show host, and where questions pertaining to organizational life were discussed in depth. The structure of the discussion was construed based on reflective teams, i.e., two actors performing a dialogue (talk show host and guest) and a silent group (audience) as listeners. The key research question concerns in what ways such an action research process is replicable? Applying a critical lens, we argue that even if strong claims of replicability are not met, as in being able to reproduce results and/or generalize them, this is outside the point. Rather, as we set out to apply a qualitative research design to achieve cogenerative learning effects, we advance an understanding of replicability-as-recoverability. This entails giving explicit grounds for our epistemic anchoring in critical realism and sketching out a research design which is sufficiently clear and transparent to undergo critical scrutiny.

## Introduction

Does an experimental qualitative research process, structured around serious play and reflective teams (RTs), satisfice claims of replicability? In this article, we investigate and scrutinize an action research process in terms of the question of replicability. We contend that our research design as such does not satisfice claims within the traditional understanding of replicability, as in reproducing similar results, where “similar” adheres to notions of ontological objectivity. However, we argue that it does satisfice a stronger claim than that of being merely *plausible*, which Checkland and Holwell (1998) argue may be “*the greatest lacuna*” in this kind of research. To this end, we investigate how and in what ways an action research design, structured around “serious play,” is *recoverable*, thus advancing an understanding of replicability-as-recoverability.

A recent stream of organizational research uses the term *serious play* to describe situations in which people engage in playful behaviors deliberately with the intention to achieve work-related objectives (Statler et al., 2011). Serious play has further reached the field of organizational research, as experiments have attempted to utilize serious play to generate new ideas, shared meaning, and deep commitment in strategy as well as to fuel scenario development processes.

In a pursuit to argue for replicability-as-recoverability, we present an action research design crafted for the purpose of exploring how various modalities of serious play may be used as methods of qualitative organizational research. Specifically, the article investigates how serious play may facilitate “unmuting,” representing the forming of new language games and the vocation of complex organizational issues which are (otherwise) easily missed, concealed, or actively kept from being voiced. Driving our research is an overall purpose to experiment with, and design, a sensemaking-arena in which researchers and participants may form a *cogenerative learning relationship* from which all parties stand to gain knowledge and insights (Elden and Levin, 1991; Greenwood and Levin, 2007). Our analysis suggests that a play-based action research design such as ours promotes the formation of new language games, which again contribute to insights and understanding of complex organizational issues, both for the participants and for researchers.

As this special issue has replicability as the topic of interest, we will discuss further how, in what ways and to which interest, our research design is possible to replicate.

### What Is Replicability and Why Does It Matter?

In the present article, we present an action research process, aimed at cogenerative learning, where researchers developed and learned together with the research subjects. The key research question concerns how such a research design may be replicable? A traditional understanding of replicability suggests that it is closer to a quantitative assessment than qualitative exploration. Replication means that research data should not only be reproducible, but also that findings ought to be generalizable. “Authenticity” rather than reliability is however often the issue at stake in qualitative research (Silverman, 2001) which entails for qualitative studies that a *valid interpretation* of the data may be more important than an exact replication producing the same findings. Silverman follows up saying that even if reliability may be achieved in qualitative interview studies by applying, for instance, fixed-choice answers and inter-rater reliability checks, it often comes at the expense of a diminished explorative approach. A strong claim of replicability is akin to a positivist perspective, where it is commonplace to distinguish between a singular, existential, and a universal proposition. Popper (1959) explains that a singular proposition can be “*a single swan is white*,” an existential proposition can be “*some swans are white*,” and a universal proposition can be “*all swans are white*.” Universal propositions, thus, assume the character of being normative (Johannessen, 1992).

Replicability in the positivist tenet makes claims of ontological objective truth by way of verification, a venture Popper famously refuted in favor of what arguably became his prime invention: the principle of falsification. Following the reasoning of falsification, one cannot hope to verify any hypothesis of truth “as such.” One can only hope, and try, to falsify (counterprove) it by excluding its antithesis (null hypothesis). (If all swans are white, then a black bird cannot be a swan, thus the empirical finding of a black swan would equal a falsification of the hypothesis “*all swans are white*.”) Replicability used as a means of verification would fall victim to the same rationale, and thus be most apt in research designed to exclude null hypotheses (i.e., falsification). It follows that the epistemic status of replicability in any research design will be a function of the researchers’ talent to produce bold and testable statements, and of testing them rigorously. The reliability of quantitative assessments would as a consequence, paradoxically, be prone to qualitative judgment. Replicability, no matter the research tradition or epistemic canon, will as a consequence only receive scientific status as judged in relation to the standard of métier set by the researchers involved.

In the present study, we take a different stance – as our aim is not to produce and analyze research data or results alone. Rather, we adopt a different role, performing research in a learning partnership *with* the research subjects (as opposed to doing research *on* them). We deploy a design of action research as a collective enterprise between researchers and research subjects into learning and developing knowledge, commonly referred to as “cogenerative learning” (Greenwood and Levin, 1998), which in turn makes the question of replicability less relevant in terms of reproducing test results claiming ontological objective value (Dehlin, 2008). The research process described in this article is indeed replicable, and may produce similar results when repeated, but there are no claims made as to the results’ and findings’ ontological objective status. Rather the focal point of the results and findings here is their epistemic status as tools of knowing, always bound to context and history, as is common within the sort of process thinking to which we subscribe.

The study is conducted on the basis of a processual epistemic view on reality (i.e., process thinking), something which means we are less concerned with ontological objective questions of “what reality *is*” than with “how it is that reality *becomes*.” Chia (2002) advocates that “*reality is always heterogenenous and becoming*” thus change in this perspective is integral to social structure (Carlsen, 2005). A process view challenges a positivist perspective on replicability, in that the concept of reproducible results presupposes a research context which is fixed and stable. As our research design is aimed at understanding the fluent and qualitative phenomenon of cogenerative learning, a process view is therefore beneficial as it provides an epistemic foundation to frame and focus (experimental) research activities.

Concretely we define our process perspective in lieu of *critical rationalism*, where a central issue is that the construction of knowledge of objects is based on the practical meaning and function they offer to us (Heldal, 2008). For example, what defines the meaning of a bridge is its presumed *practical function* to connect two opposing strands. Two important points can be drawn from this: (1) some phenomena may not be “observed” as such, only their traces or influence on observable objects; and (2) our observations may only observe what has been in the past: all observed things are influenced by the observer. In short, as an action as a whole can only be understood after it is performed, this understanding has an influence on the action itself (Heldal, 2008). Fleetwood (2005) sums up the key elements in critical realism as follows:

• **Entities can exist independently of identification:** Something may exist without someone observing, knowing, and constructing it.
• **There is no theory-neutral observation:** No unmediated access to the world is possible; it is always colored by something – be it politics, theories, etc.
• **Reality:** For critical realists, an entity is real if it has causal efficacy, has an effect on behavior, or makes a difference of sorts.

For our research design, the last point is critical. While not subscribing to any traditional perspective of replicability (as an ontologically causal outcome or result), we believe that a claim of “reality,” or rather “reality in the becoming,” is essential. For our purpose, this entails an understanding of a research process as a difference that makes a difference (LaTour, 1993), a concept we return to later, addressing what Checkland and Holwell (1998) call *recoverability*. In their research, action research is not aimed at reproducing results, but as at producing knowledge which is stronger, more structured, and fixed, than denoted by “plausibility.” To this end, they employ the term *recoverable*, which presumes starting out with a clear and declared epistemology (p. 18). In short, to achieve recoverability researchers are obliged to give a clear statement as to their epistemological anchoring – the paradigmatic understanding fueling their research design. This is what we will turn to next, after giving an account on how we methodologically applied “serious play” to construct a research design for the purpose of “unmuting”; giving a voice to those who do not have one.

### Toward an Arts-Inspired Research Experiment

Of special interest to us is the premise that artistic undertakings may serve as a window into organizational members’ tacit “knowing in your gut” (Taylor and Hansen, 2005), allowing them to grasp and communicate about things they cannot with words alone. By way of alluring to the esthetic dimension of human reasoning, the arts have the potential to collapse the cognition/emotion dualism, and as such, the arts offer sensemaking tools that may tap into domains at the “fringe of awareness” (Sandelands and Buckner, 1989): deep-seated aspects of the mind where cognitions cannot be grasped outside of the emotional processes infusing them.

Particularly, in our design, we have been looking for features to construe a sensemaking arena, which were not necessarily arts-based in a conventional sense of the arts, as exemplified by established varieties like dance, music, sculpturing, imagery, and so forth. Rather we have been looking for playful and esthetic elements, still relevant for the arts as they would invoke emotional dimensions of knowing (Schiuma, 2011), but which would carry fewer characteristics of artistic elitism, providing a lower threshold for practitioners and researchers without needing to deploy artistic expertise. “Play” is key to our experiment in that it can both allude to Dissanayake (1995), and at the same time emotionally charge, a sensemaking process so as to spur interesting and relevant reflections in a positive, trustful, and relaxed manner.

We will start by introducing play as a concept and elaborate on the idea that play may have very serious connotations and implications for organizations. Next, we present our research experiment and explain key elements in our design before we end with a discussion on how our experiment supports why and how play can be used for purposes of both research and organizational development.

### (Serious) Play in Organizations

Play is inherent in Wittgenstein’s (1994) seminal work *Philosophical Investigations*. In the opening paragraphs, he expounds how the very writing of the text had to be in the form of remarks, as he had great difficulty forcing his words into a linear narrative against their “natural tendency.” In itself, Wittgenstein’s rhetorical grip may be read as a play with orthodoxy and scholarly rigidity, much like “natural tendency” may be associated with the inescapable, yet understated, playfulness of a creative mind (such as his). On a more explicit level, the significance of play is reinforced by Wittgenstein’s introduction of the “language game” – a metaphor for the process by which a child learns to master his mother’s tongue as well as for the larger system of social (inter)actions into which language is woven (pp. 15–16).

Following Wittgenstein, play is intrinsic to the organizing of an intersubjective reality, and a work setting should be no exception. Not to say that such joint organizing of worlds is by necessity “fun,” but nonetheless playful in the sense of coming into existence through curious investigations and open-ended creativity. Paraphrasing Heidegger (2002), human beings are “always already” in play, as play is an expected feature of the unpredictable and probing wanderings of the creative mind: both as a *way into* language games and as a way of *mastering* and utilizing them in everyday situations. Interestingly, this idea receives support from contemporary neuroscience in that the working of the human mind is different from the way machines mindlessly repeat defined cycles and is more like a (re-)generative dynamic (Eagleman, 2011). Notably, play has even been depicted as essential for the survival of primates (Brown, 1988).

Play has been associated with childhood, studied as part of children’s developmental psychology (Brown and Vaughan, 2010). Taking a pedagogical route (Dewey, 1938), points out that playing a game is for children anything else than pure chaos and chance. The kind of social sense-making displayed on a children’s playground, for instance, is as much an organizing of worlds as it is chaotic make-believe. To the extent “play” constitutes “learning by doing,” an approach to integration into adult life, it ultimately enables the child to take the role of the generalized other (Mead, 1934b): Games are brought to life by merging the rules of the past with the context of the present. Sometimes rules play a dominant role in children’s games, and sometimes they are challenged, thwarted, or even abandoned all together. Playing a game, then, involves a negotiation between the orthodox and the spontaneous, and as the game evolves into new stages by way of improvisation or serendipity, the past loses its (in)formative power: Where some games are all about *not* breaking rules, call them “games of orthodoxy,” others are about continually making new ones, call them “generative games.”

In response to a shallow characterizing of play as a merely frivolous, pointless human activity, and to that effect only marginally relevant to organizations, *serious play* reflects an attempt to highlight intentionality and direction (Statler et al., 2011). To the extent “play” is considered an activity defined (and judged) by way of its intrinsic value, adding “serious” implies morphing play into an instrumental exercise, leaving serious play something of an oxymoron: Merging non-intentionality with intentionality within the same concept, is paradoxical to the extent “intrinsic” is treated as binary to “instrumental.” Moving from a nominal definition of play as intrinsically non-intentional to a pragmatic definition (e.g., Arjoranta, 2011), however, opens up the possibility that intentionality is less an either/or phenomenon than it is a gradual, nuanced, and complex intermeshing of pre- and post-rational agency, of prospective and retrospective sensemaking (Weick, 1995).

While play may be integral to the workplace, we are cautious to point out that play can be mutually constitutive, rather than antagonistic, to seriousness. A possible implication is the instrumental concern that play itself can be utilized for specific purposes, including qualitative organizational research.

### From an Epistemology of Play to a Methodology of Play

In this section we give grounds for why and how methodological unorthodoxy in the form of playful research arenas may be expedient for the purpose of qualitative organizational research. We show how play may function as a generative tool in a learning dialogue around what (Taylor, 2002) has inspired us to label “muted topics” –aspects of the workplace residing “beneath the organisational surface.”

The epistemological approach to humans as being always already in play is taking the concept of serious play a step further from the mere instrumental sense restricted to organizational development (Statler et al., 2011). Further, there may be a potential to tap into play for purposes of research by way of “installing” generative language games intended to spur open-ended reflection whilst accentuating play in the process: If play is a constituent to reality construction, it may be modeled and used in a serious attempt to get access to qualitative and embodied domains of knowledge, which are hard to tap into using research techniques where the researcher is either: 1. trying to downplay her influence on the informant to the greatest extent possible and/or 2. concerned with mere cognitive contents and thus disregards emotional and esthetic aspects of data generation. For instance, in structured interviews, the researcher may ask questions in order to get responses akin to cognitive dimensions, implicitly positioning knowledge *contents* as separate from, and for research purposes more desirable than, the *processes* of embodied knowing from which they emerge. In that sense a structured interview is less devoted to the way respondents answer than in the words they are using. The same goes for unstructured or semi-structured interviews to the extent they are aimed at explicit cognitive contents. Less structural rigidity or strictness may make these more attuned to esthetic domains of knowing, as they may allow more time and possibility for open creative association and elaboration. Specifically targeting esthetics and playfulness may go even further.

Our design is aimed at esthetic modalities of sensemaking, and it attempts to disrupt established cognitive reasoning and encourage a playful creative way of thinking and communicating. Rather than merely asking questions in a familiar manner, in a familiar setting, so as to facilitate the abduction of chains of thought burnt into the circuity of habitual reasoning, we seek tacit contents at the fringe of, or alien to, standard vocabulary.

The suppression and displacement, deliberate or not, of particular groups, sentiments, or ideas represent the “why” of our choosing our topics of research, and it also signifies why we, on a larger scale, wanted to investigate how esthetic manipulation of conversations (i.e., play) may contribute to new language games and new insights. Thus, in the following, we present a research design where play and esthetics are placed in the foreground, admitting them to infuse all aspects of data-construction. Attentive to how emotions and esthetics shape cognitive reasoning, we investigate how a qualitative design based on serious play may be apt to address the unspoken, but nonetheless felt and experienced; using play and esthetics as a means to give voice to “those who do not have one,” and contribute to cogenerative learning.

## Materials and Methods

### Muted Topics in Organizations: Finding Informants

Given that organizations are construed on the grounds of a myriad of language games, playing any organizational game involves an infinity of esthetic modalities, some more explicit, others more tacit and hidden, but all of them constitutive of (and features) of meaning. Some of the games played out in everyday organizational life are non-controversial, easily understandable, and equipped with a standard set of (verbal) rules, signs, and protocols, perhaps even with a formal jargon of its own. Examples include standard issues of bureaucracy and technocracy. By way of given rule-sets, routines, and procedures, in these cases a language is already installed, to a large extent facilitating mundane conversations to take place – a language that is easily understandable by those familiar with it, equally difficult for those not included.

In some instances of everyday work, however, actors are involved in problems or situations where a preinstalled formal or standard language game is lacking. This concerns aspects of everyday organizational life where issues fall outside of defined organizational borders and structures and outside of established bureaucracy and technocracy and where problems are more complex than they are complicated (see Dehlin, 2012). Power dynamics, for instance, is prone to create complex situations that are both hard to understand, difficult to cope with, and sometimes hard to communicate around (see for instance Brown and Coupland, 2005). Inspired by Taylor’s (2002) “aesthetic muteness,” we suggest labeling these hidden, complex aspects of organizations “muted topics.” These are not just esthetically mute to the extent that they are hard to address from an esthetic perspective (Taylor, 2002). On a more general level, they may represent somewhat explicit issues, but to which there are few established communicational channels, and with the possible implication that important “viewpoints” are displaced, missed, even suppressed, and effectively left unvoiced.

In organizations some groups, competencies, or professions are *acknowledged* and *recognized* over others. Our interest concerns the skewed distribution of recognition between those who directly contribute to the value-chain of organizations and those who merely provide support services. We have worked from a hypothesis that administrative personnel in Higher Education (HE)-institutions may be representative of such a group of professionals that are acquainted with recognition and particularly the lack thereof, to the extent that they merely provide support to core activities rather than performing them. As this group is formally positioned external to the value chain, it makes them suitable as informants.

Our research group of four scholars are all employed by the same university, three at a business school, the fourth in the educational sciences. In our experience as HE-employees, administrative staff in HE seems to be at the receiving end of a very modest, if not scarce, amount of attention and recognition in everyday organizational life, whereas most formal recognition is attributed to the quantifiable achievements of research personnel and to the practices of research and teaching. Add to that what we see as an apparent lack of focus on administrative personnel in contemporary leadership research, sketches of an empirical experimental design emerges, by way of a combination of giving voice to a “voiceless” group of professionals and experimenting with new ways of generating qualitative data.

As to where to go to find informants, the sheer unconventionality of our design felt so risky we recognized the need for experimenting in a setting that allowed us to feel safe. We came to realize that the business school campus could be considered a safe location for serious play and decided to target its administrative staff. We already knew them well, and a bond of trust and mutual confidence, we believed, was already installed. Their immediate acceptance to participate and the positive sentiments communicated accordingly, supported our belief. The research set-up was thus a combination of researchers (the authors), and research subjects (the administrative staff).

### Designing Play for Research

“Enclaves” (Friedman, 1993), “interspaces” (Antal and Strauß, 2013), “transitional spaces” (Küpers, 2011; Winnicott, 1971), and “fabulation” (Flaxman, 2012) exemplify social arenas that allow people to play with ideas, roles, and identities in a relaxed, non-threatening atmosphere. Our research design is an experiment in concretizing such an arena based on the premise that esthetic expressions can be mediating tools when inviting researchers and informants as equal partners in a learning conversation (Darsø, 2008; Klev and Levin, 2009). Our design is a combination of “role play” (Role play, 2017) and “reflective teams” (Andersen, 1987). A *role play* is a setting where people assume certain attitudes, actions, and discourses in a make-believe situation to understand a differing point of view or social interaction (Role play, 2017).

As a role play we have chosen a TV talk show – a television programming genre in which people of significance/influence, often authorities in a particular field, participate in a discussion facilitated by a talk show host (Talk show, 2016). We labeled the role play “Skavlan,” after a famous talk show in Norway. Skavlan is recorded in front of a live studio audience in Stockholm and in London, and is broadcast during prime time on Friday night in Norway and Sweden throughout the show’s season (Skavlan, 2016). During the show, host Fredrik Skavlan has “in-depth and earnest interviews with some of the world’s biggest stars, artists, politicians, scientists, writers, and philosophers” (Skavlan, 2016). The show is structured around the host’s conversation with the guests and the guests’ conversation with each other. We chose this angle on play as it is a familiar setting to the participants.

Further, we added a subsequent reflective process (Schön, 1983) with the intent to achieve even deeper reflections and conversations, and spurring further intersubjective anchoring and substantiation. We chose a “reflective team” (Andersen, 1987) as a social sensemaking tool to complement the more playful and artistically inspired Skavlan setting. The unmuting potential of the RT and the likelihood of it bringing about new perspectives were qualities that made it suitable in two regards: firstly, as a means to debrief and validate the talk show as a research method from a participants’ view, and secondly, content-wise, to permit an even deeper reflection on the significance of recognition in the workplace.

Rennemo (2006) defines a RT as a group of people who come together to talk about someone they have observed in action. It is inspired by the “Milano-model” that psychiatric doctor Tom Andersen developed into a concept throughout the 1970s and 1980s as a systemic way of facilitating authentic dialogue with an emphasis on listening and turn taking. It is beyond the scope of this article to provide a thorough review of RTs, but it is noteworthy how the method has spread from the original therapeutic context and is now applied in a wider organizational context (Hornstrup et al., 2005).

Arguably, an RT is not role playing as much as it is a facilitated process of turn taking, something which makes it suitable for our need for a debriefing and knowledge expanding tool connected to the preceding serious play with the TV talk show genre. Coerced turn taking renders the RT to some extent a serious play with the structures of more orthodox group interview techniques. Of particular importance for us is how RTs involve participants conducting private dialogues with other interested parties listening in on their conversation, upon which roles are switched. Not being granted the ability to enter the dialogue while maintaining the role of a listener forces the listener to pay attention to others rather than to (voicing) one’s own opinions. In other words, in reflective processes, transitional space (Winnicott, 1971; Küpers, 2011) is consciously created for the purpose of maintaining a natural flow and pace of conversation for those engaged in dialogue without the peril of interruption. For instance, in the case of the abusive father listening in on his family’s conversation around the father’s abusive behavior, cut-off from the ability to break in on it (Andersen, 1994), space is created that allows mutual (novel) understanding to arise for the father and the family members. To the extent that this transitional space is built up by various forms of emotional-cognitive tension, it makes for a suitable object of study from an esthetic angle.

Having presented major concepts of our arts-inspired research design, we now turn to a fuller presentation of how (and why) we designed *Skavlan* and the *reflective team* as a research experiment.

#### Data Collection

Two months before the actual experiment we e-mailed two senior managers in the administrative staff, informing them about our plan to create a pilot study in which we could conduct data collection experiments. We shared our reflections on administrative staff as a “voiceless” group in organization studies and our interest in developing better insight into their daily tasks, motivational aspects, and professional values. We introduced the idea of using Skavlan as a research concept, asking the managers to make inquiries about the willingness of staff to participate. We got our first reply in less than 25 min, and an enthusiastic one at that: “Yes, we would very much like to play Skavlan with you! This sounds very interesting!” The next reply added: “Voiceless group? Yes, that is perhaps a good way to describe this large professional area.”

The two managers jointly suggested arranging for a meeting with the entire administrative staff, not least because they worried some of the staff members would be skeptical about the whole idea. As it turned out, however, most of the participants responded positively to our concept, whilst only a few remained hesitant. Since we emphasized that participation was voluntary and suggested that candidates for the role play could have a say in the casting of roles (guest or audience), we were given the signal to move forward. Table 1 gives an overview of the manuscript in terms of activities, participants, and roles.

**TABLE 1:** Overview of the Skavlan session.

Talk show session	Activity	Participants and roles
Time scope: 1 hour Where: A campus pub	**TV talk show set up in a mock studio with live audience**	Seven administrative professionals (APs) participants One researcher as talk show host Participants from research team as observers Camera man (a representative from the technical staff)

Time scope: Half hour	**Reflective team**	Roles: • Reflective team: APs from the talk show audience • Facilitator: Researcher

Time scope: Half hour	**Plenary reflection**	• All APs and researchers • Facilitator: Researcher

### The Skavlan Talk Show

When all technical details were on track, the host (a representative from the research group) entered the stage, giving the cue for what is for Scandinavians a well-known vignette. Next, the host introduced the guests by imitating gestures and phrases from the authentic Skavlan: Each guest receiving a tailor-made celebrity-like introductory speech intended to reflect respect, recognition, and individual attention – all with a warm humorous undertone. We include the following example:

She is the kind of person you ask for help when in need for moving a sofa or a mountain. Add to that her love for bungee jumping and roller coasters, and that she’s well known for high-speed sledge driving, we see the outlines of a strong thrill-seeking hard-working woman from [Name of home town]. She is your typical home sweet home-farmer girl who currently works as a senior advisor in the urban capital of [name of region]. Please, welcome [Name guest 2]! (applause)

**FIGURE 1:**
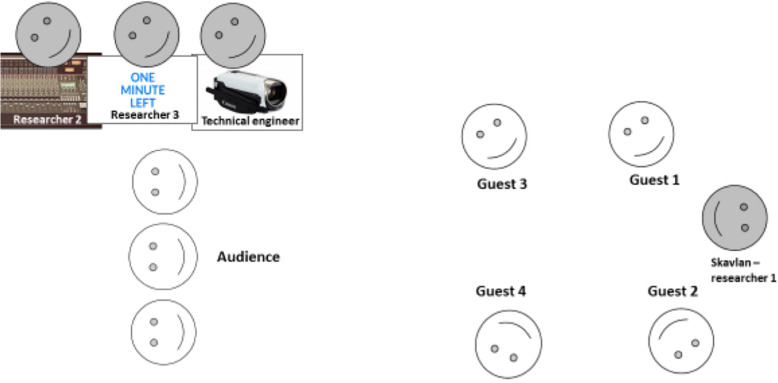
The Skavlan TV talk show session scene.

Next, applause was directed by a representative from the research group posing as a TV-show crew technician, instantly spurring laughter across the audience, see Figure 1.

Upon presentations and rounds of applause, each guest was given a 10-min exclusive with the host structured around the following questions:

1. What are the three most important missions that the business school performs?
2. Name the top values that form the basis for your doing a good job?
3. Describe the ways in which you receive recognition for the job that you do, and explain from whom you receive it?

This entire routine was repeated for all four guests, and in contrast to the original Skavlan concept, which focuses on both Skavlan’s conversation with the guests, and guests-to-guest conversations, we made a decision to focus on Skavlan’s sequential conversations with the individual guests only. As such, we were aiming to stage for an “ideal speech situation” (Habermas, 1970) where individuals are free to communicate openly, free from distortions of power. The one-to-one structure is an impersonal, non-negotiable routine that monitors “air time,” ensures that every individual has the chance to solo, and offsets influential participants who might otherwise dominate a conversation (Barrett, 1998). Moreover, whereas in the original Skavlan only the most prominent guests are allowed to sit down during the entire show, we chose to let all our guests be present on stage during the full session.

#### Reflective Team

As the host ended the talk show, characteristically throwing the cue cards over her shoulder, the vignette sounded. A member of the research group directed applause and the fading of lights, upon which we made some small reorganizations of chairs to set up for the RT. Simultaneously, three administrative professionals got ready to shift roles from audience member to RT member.

A member of the research group led the RT session, in the way that she encouraged the participants to reflect on what they heard in the previous session and to offer their spontaneous associations, sentiments, and thoughts. The participants were directed not to address the cast members directly (who had now taken their places in the audience), but rather to talk about them as if they were not present (see for instance Andersen, 1987). The RT session lasted about 10 min, until we ended with an open, plenary reflection on experiences and responses to the foregoing events. In this last session, we made sure to ask for feedback on how the participants experienced and valued our research design. Fortunately, they took great interest in sharing their experiences with us, provided useful information, and encouraged us to pursue this line of research to explicate issues that are otherwise hard to address.

The entire session was videotaped and the contents shared and analyzed by the four researchers individually, after which the research group as a whole analyzed and discussed the data. Further analysis was made by means of open coding, asking of questions (Strauss and Corbin, 1998), the constant comparative method (Glaser, 1992; Charmaz, 2000), visual displays (Ryan and Bernhard, 2000), and theoretical sampling (Strauss and Corbin, 1998; Charmaz, 2000).

## Results

In the following section, we present the results from our experiment, structured around four main findings: Role play makes sense, deep meaning from deep listening and disciplined turn taking, unmuting issues through lingering, and new perspectives from third-person objectification. We will deal with each topic in turn.

### Finding #1: Role Play Makes Sense

By introducing some simple rules for conversation and dividing the staff into guests and audience, we accomplished several things with this experiment. Firstly, the talk show guests were given the chance to gestalt their role of their own person, to spend time reflecting on their everyday trot and the significance of their work with a talk show host, with a group of researchers and an audience taking deep interest. Even if the guests were merely playing a game, they immediately and without hesitation went into deep-seated discourses, touching on sensitive areas as well as more general issues of everyday concern. Without exception, each guest played their role with much devotion and empathy. Despite the obvious playfulness in the setting, the talk show was soon turned into a meaningful arena where serious acting ensured that make-believe became authentic conversation. A key word is “serious” – the talk show was indeed a game, but the questions, the setting, the cameras, jingles, and enthusiastic performance of the host, all contributed to authentic sensemaking. The talk show setting allowed for play and laughter, and even if the subjects discussed were serious enough, the mood in the room was generally light and playful.

It seemed playfulness was a necessary component in bringing forth genuine emotions and reflections, which again spurred intriguing sensemaking. Both participants and audience reported being “drawn into” the game, gestalting roles, laughing, acting, and sharing intimate thoughts and sentiments. The TV-talk show appeared to be sufficiently detached from everyday clutter and established language games, and it was perceived to be a safe place to shake off possible intimidation, associated with the sharing of vulnerability in front of colleagues. In their own words, the setting itself comprised a “non-threatening arena,” to lay out inner feelings and thoughts. Such feeling of safety was reinforced by the fact that Skavlan to begin with was a well-known concept for all participants, with a predictable structure and a set of conventions, all of which contributed to a successful role play.

### Finding #2: Deep Meaning From Deep Listening and Disciplined Turn Taking

In both Skavlan and the ensuing RT, the participants were compelled to listen to the conversation without the ability to interrupt, give input, or ask questions of any sort. This “having to listen to” colleagues acting as guests was arguably something that provoked and sparked meaning making, as guests were addressing issues in which the audience were already deeply vested. The guests were in a (very real) sense talking about *them*, the audience, as well as themselves.

A participant from the audience openly expressed her appreciation for what she had heard: She was impressed and baffled and said she found pride in the manner in which her colleagues chose to describe their role and work situation. She had never thought of it “that way,” she said, indicating that until now, she had not grasped the full depth of their significance as a group. She felt as part of a whole, and only now did she fully realize the vast amount of resources the administrative staff represented. She concluded that more appreciation and recognition from students and colleagues was due.

As in Skavlan, the switching and turn taking dynamic between reflecting and listening in the ensuing RT allowed for the emergence of many layers of reflection, and of particular importance, was the voicing of perspectives on recognition. In both Skavlan and the RT, then, we noticed a sort of deep listening – a listening that was focused, attentive, and tuned in, implying that something was at stake. The dialogue brought about deep, often unexpected, meaning, and the viewpoints discussed and feelings displayed, encouraged core reflections on their raison d’être as a group. There seemed to be recognition in being encouraged to reflect upon recognition.

During the talk show, deep meaning was found in voicing issues that otherwise may not have received attention on account of established conventions steering the conversation on autopilot. Aspects of lacking recognition turned out to be muted in the playing of everyday language games, but whether actively muted or passively foregone in an ever so busy work situation is difficult to deduce from our material. Important, however, is that these issues were not superficialities, but they were seen as pivotal to how the participants view their social status and their organizational identity (i.e., “who they are as administrators”).

### Finding #3: Unmuting Issues Through Lingering

Lingering itself seemed to be a valuable aspect of our design. As a result of the gameshow rules, the conversation was not allowed to derail or take on spurious, unexpected, or digressive paths, but was rather disciplined by the host to stay on track, digging deep and inviting patience and interest from all those involved. Further, according to the participants, the host’s way of “locking in” the respondent’s responses left them feeling comfortable, and it made them offer surprisingly honest answers, even if they knew they were playing a game.

Since participants were mostly used to discussing daily operations and mundane occurrences superficially and not addressing the general significance of their work with regard to a larger organizational perspective, nor the values or epistemic background for why they do what they do, the talk show provided an arena and conversational mechanisms/structures to linger on issues otherwise easily foregone. This was something out of the ordinary for a staff of administrators who usually try their best to do as much as possible during a busy work schedule.

### Finding #4: New Perspectives From Third-Person Objectification

The audience praised the rare possibility of “seeing oneself from the outside.” Mere collectivity alone may not have made this possible, but the third-person view-point built into both Skavlan and the RT allowed for sufficient distance and objectification so as to permit and encourage testing out established “truths” and possibly seeing oneself in a new light. For instance, the Skavlan audience was particularly baffled by the idea discussed by guests that colleagues and students may not really notice them in everyday work life, far less acknowledge their importance – “their greatest fear,” as someone called it. This was followed by reflections as to “who are we if not important contributors to the fulfillment of the Business School’s aims and needs?” At this point, ideas were exchanged as to how to become more salient, and suggestions included the use of informational meetings, posters, and other measures to spur attention.

The mirror-effect created by having in part of the group listen to the others speculating on “who we are” made a particularly strong impression on us as researchers, and it encouraged us to widen our perspective on recognition. Questions like “who are we” indicate that recognition is a far more comprehensive topic for the participants than, say, mere appraisal and gratitude. Widening, or rather deepening, the scope, recognition seemed to touch on aspects of their identity and existence, and the third-party view in our design seemed to bring the participants into close contact with, and facilitate a dialogue around, questions that were normally muted.

## Discussion – The Power of Serious Play: Replicable?

We have described a research process used to unmute organizational groups, which in normal day-to-day settings have little impact. The process may be summarized as follows:

1. Identify a group of people (formal positions, social groups, or communities of practice) that lack a voice in the organization (in this case the administrative staff).
2. Identify a group of people within the same organization that has voice (in this case the authors).
3. Set up a role play cast with a famous talk show (in this case it was Skavlan). It should be filmed, both as to emulate the talk show setting as well as to collect data.
4. Let the voiceless group decide who should be in the audience and who should be guests. One person from the has-voice group should function as the talk show host.
5. Set up a structured interview format revolving around recognition, meaning and identity.
6. Once the talk show session is ended, a RT session is facilitated by a member of the research team. This session should start out with open associations, feelings, and sentiments.
7. The overall session may conclude with suggestions for future steps.

In discussing the findings in lieu of replicability, we cite Checkland and Holwell (1998):

“*Achieving credibility, consensus, and coherence does not make a ‘truth claim’ as strong as that derived from replicability of results independent of time, place, and researcher. Action researchers must pay careful attention to the claim of validity relevant to their research into phenomena not ‘homogenous through time.”’.*

As much as this statement reflects the research ambitions behind this article we follow Checkland and Holwell (1998) in stressing the importance of not to withstand from claims of replicability on the basis of an unstable reality, as in some sense this will apply to all qualitative studies where the researcher is using him/herself as a data collecting instrument (Tjora, 2006). Thus, in addition to discussing claims of validity, we address how and in what ways such a research process is *recoverable* (Checkland and Holwell, 1998), something which according to Checkland and Holwell presumes an explication of the epistemic position fueling the research design.

### Paving Way for a New Language Game

An interesting aspect of Wittgenstein’s (1994) conception of a language game is the implicit and very wide understanding of the term *language* itself: Language is not exclusive to the utilization of specific verbal signs or to reified symbols of a particular sort, but it alludes to any instrument used socially to create and communicate meaning. Further, there is a strong action-theoretical component in Wittgenstein’s proposition, as a language game has to be played for it to come into existence, and its meaning, social as private, is tied to the contextually specific (inter)actions of significance constituting the game. More than it is a system or entity, a language game is on a very deep level, an activity of structuration, wherein actions may make use of explicit language symbols, such as words and numbers, but may also be of a more subtle and artistic constitution, such as a facial gesture or a musical expression.

We find that our design laid the context for an alternative language game to emerge, new to the participants, and where muted issues could be voiced and made sense of. As a language game, our design facilitated subtle conversational cues, features, and interactions, such as emotional gestures and responses of enjoyment, surprise, and disappointment, but also, on a more explicit level, new conversational rules. The design spurred a different, more playful style of dialogue allowing for authentic insights to emerge and be discussed. Sketches of a new language game could be seen as emerging to the effect that established rules and styles of dialogue at the workplace were renegotiated, even set aside, resulting in a new dialogical esthetic. This new esthetic was one of regulated turn taking, forced listening, and lingering on issues for a long enough time for insights to emerge (gradually and) socially (rather than hastily and abruptly moving on to digressions and detours). It was also an esthetics of play, wit and curiosity, and encouraging and promoting collective reflections and learning (Klev and Levin, 2009).

As this cogenerative learning was declared in advance as part of our epistemology, it seems our design collapsed a conceptual distinction between technical rationality and creative thinking, and that the former became something of a tool for the successful emergence of the latter. Open creative thinking seemed to be made possible *because of* the implemented structures, not despite of them. A possible explanation for this is the manner in which the structures were “breathed into life” (Dehlin, 2012), both by the guests and the audience but not least because of the convincing and dedicated manner in which the host played her role. There was a perceived safety in the host’s way of focusing solely on individuals one-by-one, but arguably the show setting itself, familiar to all participants, contributed to a trustful conversation. As a result, a delicate balance was achieved between psychological safety (Edmundson, 1999) (non-threatening atmosphere) and cognitive focus. This balance seemed to be cultivated with the utmost sensitivity by the participants, paving way for a fresh language game apt for unmuting tacit issues. This unmuting, we contend, approaches a claim of validity to the process.

### Serious Play: A Context for Identity-Construction

The results of our study demonstrate five important things about identity construction. Firstly, it supports a close connection between self-image, self-worth, and the image projected by others, supporting the notion that the image presented by others is pivotal for self-identity construction (Hatch and Schultz, 2002). Secondly, a negative discrepancy in the sense that others’ perception of one’s identity and social standing are devaluating in comparison to one’s self-identity, creates a tension and the eliciting of negative emotions. Thirdly, our analysis implies a close connection between a self-induced identity and the larger organizational identity, as shown by the manner in which the participants individually identified with their team.

Fourthly, relations between personal and work identity remained for the most part a mute issue for which a suitable language game arena was not installed. Explicating, reflecting upon, or making sense of the way they felt unrecognized was hard if not impossible. The non-existence of a language game implied there was no set of rules, no grammar, and hence no conversational processes to address identity issues related to recognition, though these nonetheless appeared important for the participants on an everyday basis. The fact that identity issues for the most part remained muted topics comprised a problem in itself, let alone the negative discrepancy between self-induced and other-induced identity. In that regard, our serious play provided a tool and an arena for unmuting, and it marked a starting point for conversations around identity that would turn out to continue for months. As a part of meaning construction, this element of identity construction is a social venture (Mead, 1934a; Weick, 2001). The development of the self is largely dependent on a process of changing with “*the other*” (Mead, 1934a) – which attests to a change also with the researchers in the study. Our design facilitated an honest and non-threatening venture into a complex cognitive, social, and emotional terrain – all of which seemed intertwined and inseparable as different aspects of a larger process of identity construction. We believe that staging a playful arena, using comedy and wit, made tensions easier to address and handle.

### The Talk Show – Recoverable?

Our research experiment was designed to investigate themes rather than testing hypotheses (Checkland and Holwell, 1998), and the contents of our findings is akin to the action research producing them. The theme of unmuting has been important from the top, but not as a hypothesis: We wanted to learn how and if a cogenerative learning process could be staged so as to elicit and give voice to those normally not voicing up (for various reasons).

We follow Checkland and Holwell’s (1998) suggestion in withstanding any “strong criterion” of replicability (for instance repeatability), but at the same time we claim to be more stringent than the weakest of criteria of replicability (for instance, that the research story is “plausible”). This, in their view, entails employing in advance a clear and declared methodology (which we have accounted for), and a process sufficiently clear and transparent so as to allow and invite critical scrutiny (p. 18). Among other things, this implies an attention to which parts of the process account for what counts as knowledge. We believe our walk-through of the process, coupled with possible interpretations in the previous section of the discussion, in that regard is satisfactory.

We believe that the sketched process is a sound way of giving voice to those who are not empowered or recognized in formal power structures. As such, and to this end, replicability can be denominated as a recoverable process and not merely as a measure of reproducing generalizable results. As we advance an understanding of replicability-as-recoverability, it allows for an extension of research methodology from the science domain into the larger organizational sphere, thus contributing to bridging the research-practice divide and allowing for the advancing of knowledge on both ends.

### Implications for Research

Separately and together the talk show and the RT seem to be effective ways of generating data on muted issues. They seem very apt as arenas for sharing and reflecting, but also for generating new ideas and learning for those participating – both as “informant,” which becomes more of a cogenerative sense maker than a mere source of information, and as researchers, who also take part in the same process of cogenerative learning (Elden and Levin, 1991). It seems the introduction of new, playful, but still sufficiently coercive conversational rules spurs deep listening and regulates turn taking in such a way that the setting promotes a unique, engaging dialogical dynamic.

### Implications for Practice

In organizational life, the appropriate language game for communicating complex issues may not be readily at hand. Consider the instance of equivocal feelings and esthetic experiences: Vague sentiments, incomplete thoughts, and conflicting attitudes or values are by their nature hard to pin down and even harder to communicate to others. The same goes for conflicting perceptions, interests, and power plays, which are cognitively tentative and as emerging matters always incomplete, partial, and shifting. In a hyperbolic strength-based work environment (e.g., Rath and Conchie, 2008), for instance, there may be lack of language games in which to participate to communicate weaknesses (Kaplan and Kaiser, 2013). Similarly, in a hostile, negative work environment, dominant language games may displace creativity and the potential for free expression so as to produce an evil, self-fulfilling spiral of efforts countering learning and development (Argyris, 2003). As language games in the workplace over time may tend to value orthodoxy over contemporariness, with the result that conservatism supersedes free-thinking, established rules of conduct, work jargon, and power plays may severely limit accepted and feasible channels of organizational communication.

von Hippel and Trivers (2011) state that an idealized organizational image, structure, or system may be instated by managers to which effect organizational members are stripped of their social status. Under such circumstances, not only the channels of communications may be constricted, but also the manner in which communication is regarded as “proper” (or not) as well as the very topics up for debate – including which organizational groups are regarded as being in a position to promote them. This amounts to a sort of work situation where not only particular sentiments are suppressed, but where some ideas, everyday conversations, and organizational behavior is displaced, thwarted, and skewed, and where established language games are used as tools to maintain the *status quo* by, for instance, the deployment of organizational defense mechanisms (Argyris and Schon, 1978). Reifying particular language games, giving them prominence and hegemony, may hamper critical thinking as much as authentic feeling, creating cultural conservatism where significant cognitive spaces are left unaddressed, even suppressed for the sake of preserving *status quo* (see for example, Weniger, 1953; Halvorsen et al., 2016). We have chosen to label these “muted issues,” and they may include spaces with potential for expressive freedom, learning, and novelty that are made unreachable, upon which routines are established to protect existing theories of action (Argyris and Schon, 1978), and yet other routines are put in effect to ensure this unreachability is maintained (Argyris, 2003). We suggest that a design like ours, being crafted from a principle of replicability-as-recoverability, can go a long way in providing an alternative language game with the potential to channel and voice muted issues.

## Conclusion

In this article, we have investigated how, what, and why an action research process structured around “serious play” for the purpose of “unmuting” is replicable. Firmly leaning on process thinking, as explicated in the epistemic tradition of critical realism, we have deployed the framework of Checkland and Holwell (1998) to advance the proposition that replicability can be defined as recoverability, something which deviates from any “strong criterion” of replicability as reproducing similar results or outcome. As our research design is reproducible, however, it may generate cogenerative learning effects transcending and potentially bridging divisions between the researcher and his subject, advancing knowledge for all the parties involved.

## Data Availability Statement

The raw data supporting the conclusions of this article will be made available by the authors, without undue reservation.

## Ethics Statement

Ethical review and approval was not required for the study on human participants in accordance with the local legislation and institutional requirements. The patients/participants provided their written informed consent to participate in this study.

## Author Contributions

All authors have contributed in designing the experiment, analyzing the results, and writing the manuscript. FH had a major role in writing the finalized the manuscript, analyzing the results, and singling out contributions and conclusion.

## Conflict of Interest

The authors declare that the research was conducted in the absence of any commercial or financial relationships that could be construed as a potential conflict of interest.
